# The effect of cigarette price increase on the cigarette consumption in Taiwan: evidence from the National Health Interview Surveys on cigarette consumption

**DOI:** 10.1186/1471-2458-4-61

**Published:** 2004-12-14

**Authors:** Jie-Min Lee, Tsorng-Chyi Hwang, Chun-Yuan Ye, Sheng-Hong Chen

**Affiliations:** 1 Department of Logistics Management, National Kaohsiung Marine University, Kaohsiung, Taiwan; 2 Department of Applied Economics, National Chung Hsing University, Taichung, Taiwan; 3 Department of International Trade, Overseas Chinese Institute of Technology, Taichung, Taiwan

## Abstract

**Background:**

This study uses cigarette price elasticity to evaluate the effect of a new excise tax increase on cigarette consumption and to investigate responses from various types of smokers.

**Methods:**

Our sample consisted of current smokers between 17 and 69 years old interviewed during an annual face-to-face survey conducted by Taiwan National Health Research Institutes between 2000 to 2003. We used Ordinary Least Squares (OLS) procedure to estimate double logarithmic function of cigarette demand and cigarette price elasticity.

**Results:**

In 2002, after Taiwan had enacted the new tax scheme, cigarette price elasticity in Taiwan was found to be -0.5274. The new tax scheme brought about an average annual 13.27 packs/person (10.5%) reduction in cigarette consumption. Using the cigarette price elasticity estimate from -0.309 in 2003, we calculated that if the Health and Welfare Tax were increased by another NT$ 3 per pack and cigarette producers shifted this increase to the consumers, cigarette consumption would be reduced by 2.47 packs/person (2.2%). The value of the estimated cigarette price elasticity is smaller than one, meaning that the tax will not only reduce cigarette consumption but it will also generate additional tax revenues. Male smokers who had no income or who smoked light cigarettes were found to be more responsive to changes in cigarette price.

**Conclusions:**

An additional tax added to the cost of cigarettes would bring about a reduction in cigarette consumption and increased tax revenues. It would also help reduce incidents smoking-related illnesses. The additional tax revenues generated by the tax increase could be used to offset the current financial deficiency of Taiwan's National Health Insurance program and provide better public services.

## Background

One of the problems in controlling tobacco in Taiwan is that cigarette prices are lower in Taiwan than in most countries [1,2]. In India, smokers have to work 77 minutes to afford a pack of cigarettes, in Indonesia 62 mins, in China 56 mins, but in Taiwan they need only work 7 to 10 mins to afford a pack [1]. As long as the domestic cigarette price remains rather low, we probably will not see much of a decrease in the number of smokers.

The smoking population increased to 4.5 million persons (total population 22,520,776) in 2002 [3]. One out of three adults smoked. Currently, due to illnesses and death associated with cigarette smoking, smokers currently account for approximately 20 billion NT dollar of extra medical expense annually and account for a 160 billion NT dollar loss in GDP (Gross Domestic Product)[4]. This economic burden in putting pressure on the government to further increase the existing Health and Welfare Tax on tobacco.

Because high excise taxes on cigarettes have been found able to reduce cigarette consumption [5-8], such measures are becoming one of the most important means of controlling tobacco [9-11]. The new tax scheme enacted on 1 January 2002 in Taiwan resulted in NT $16.8 tax excise. This tax included the existing taxes for NT $11.8 and a NT $5 Health & Welfare Tax for a 20-pack of cigarettes. The government also levies 5% sales taxes. Under that tax scheme, the cigarette tax revenues account for 40% of the retail price, which is about NT $42.2. While 40% sounds high, it is actually lower than the taxes imposed on cigarettes in developed countries that have seen some success at lowering cigarette consumption.

The government should take elasticity of demand into consideration when deciding whether to increase or add an excise tax levy. If there is a price elasticity below 1, a tax increase brings about a decline in consumption and an increase in total tax revenues. Since Hsieh, Hu and Lin (1999) found that figure to be -0.6 in Taiwan, it can be reasonable to assume that a tax increase would be more likely to reduce cigarette consumption more significantly in Taiwan than in other countries with lower price elasticities at least in the short-term and medium term [12]. Higher taxes would also generate higher total tax revenues.

Taiwan's Tobacco and Wine Tax Law is currently under review in the Legislative Yuan of Taiwan. Some legislators are seeking to increase the Tobacco Health and Welfare Tax by NT $3 per pack, raising it from NT $5 to NT $8 per pack. Just as they have done in the past, cigarette sellers will probably shift the tax increase to the consumers letting them be responsible for the increase. The effect of the price increase on demand depends on cigarette price elasticity – the larger the elasticity, the larger the reduction in consumption. Therefore, an estimation of price elasticity for domestic cigarettes could be a very important indicator of the possible effect a "Tobacco Health and Welfare Tax" would have on cigarette consumption and could be used to adjust Tobacco Health and Welfare Tax accordingly.

Price elasticity of cigarette in Taiwan has been mostly estimated using time-series data [12,13], though this method might overlook the impact of smuggled cigarettes on the price elasticity and overestimate the price elasticity. It might be more appropriate and useful to use cross-section data from the Health Interview Survey to estimate the effect of cigarette tax on cigarette consumption and to compare differences in cigarette price elasticity with various smoker characteristics.

## Methods

This study uses data on current smokers from 17 to 69 years of age during years 2000 to 2003. First, the demand function of the current smokers was established. Respondents who answered "everyday" or "some of the days" to the question how often you smoke were classified as "current smokers". Then, we used a random sampling of how they consumed and how much they paid for it between 2000 to 2003 to calculate cigarette price elasticity. We then analyzed differences in cigarette price elasticity in smokers categorized according to gender, age, education, income standard, and how much they smoked.

### Demand function

Cigarette demand function was estimated by OLS and expressed by double logarithm function. The estimated demand function we used was:

ln *Q*_*ig *_= *α*_0 _+ *β*_1 _ln *P*_*ig *_+ *γ*_2 _ln *I*_*ig *_    (1)

where

lnQ_ig _is i'th current smokers' logarithm representing monthly amount smokers consumed per person in group g. Current smokers were categorized according to age, gender, education, monthly income, and amount smoked. lnP_ig _is i'th current smokers' logarithm representing cigarette price per pack (NT$) for smoking characteristics group g. lnI_ig _is i'th current smokers' logarithm representing income per capita (NT$) for various categories of smokers in group g.

The *α*_0_, *β*_1_, and *γ*_2 _are parameters to be estimated. In order to measure how price change might affect cigarette consumption, the determination of price elasticity was particularly important. Price elasticity of demand for cigarettes is defined as the percent change in consumption resulting from a price increase. Cigarette price elasticity of demand *β*_1 _and income elasticity of demand *γ*_2 _can be derived from logarithmically differentiation (1) according to price and income.

### Data collection

Using an annual face-to-face survey on cigarette consumption from 2000 to 2003 by Taiwan National Health Research Institutes, we collected data on how many packs current smokers consumed, how much they paid for a pack of cigarettes, how much they earned per month and how much they spent on cigarettes per month. Current smokers were categorized into gender, age, education, income, and amount smoked. Calculations of cigarette price were based on the average retail price of the top 3 most consumed cigarettes, calculations of number of packs smoked per month were done by dividing the monthly cigarette consumption by the average retail price. Calculations of income were based on personal monthly income.

Certain background characteristics are listed in Table 1. More than 90% of the smokers were men; less than 10% women. The numbers of young smokers were rising at the time of the study. Young people between the ages of 17 and 24 years old made up 5.4% of the sample in 2000, while they made up 12.8% in 2003, a 1.4 percent increase. The number of elderly smokers above the age of 55 gradually declined from 15.5% in 2000 to 10.8% in 2003. People with higher educational backgrounds tended to smoke more. More than 60% of the current smokers had senior or junior high school educations between 2000 and 2002. Second only to smokers with senior high school degrees, the percentage of the smokers with college degree increased by 27.2% in 2003, accounting for almost 40% of all the smokers. Thirty-five to forty percent earned between NT $20,000 to NT $30,000 per month. Those who smoked less than one pack were defined as light smokers; 1~2 packs (2 packs excluded), medium smokers; and 2 packs and above, heavy smokers. The proportion of light smokers had gradually increased from 50% in 2000 to 60% in 2003, and the proportion of heavy smokers gradually decreased from 11.8% in 2000 to 6.5% in 2003, showing an overall tendency toward reducing consumption.

**Table 1:** Background characteristics of the current smokers in Taiwan, 2000–2003

	2000		2001		2002		2003	
				
Characteristics	No.	%	No.	%	No.	%	No.	%
**Total**	856		632		521		493	
**Gender**								
Male	789	92.2	599	94.8	496	95.0	460	93.3
Female	67	7.8	33	5.2	25	5.0	33	6.7
**Age**								
17–24	46	5.4	35	5.5	41	7.9	63	12.8
25–34	179	20.9	133	21.0	94	18.0	101	20.5
35–44	299	34.9	222	35.1	190	36.5	177	35.9
45–54	199	23.2	149	23.6	122	23.4	99	20.1
55-	133	15.5	93	14.7	74	14.2	53	10.8
**Education**								
College and above	168	19.6	128	20.3	107	20.5	134	27.2
Senior high school	317	37.0	245	38.8	195	37.4	196	39.8
Junior high school	216	25.2	153	24.2	136	26.1	98	19.9
Primary school or lower	155	18.1	106	16.8	83	15.9	65	13.2
**Month income**								
No income	98	11.4	93	14.7	63	12.1	75	15.2
<NT $20,000	146	17.1	94	14.9	94	18.0	84	17.0
NT $ 20,000–39,999	335	39.1	237	37.5	192	36.9	174	35.3
NT $ 40,000–59,999	181	21.1	144	22.8	117	22.5	105	21.3
≥ NT $ 60,000	96	11.2	64	10.1	55	10.6	55	11.2
**Smoking degree**								
Light smokers	445	50.0	319	50.5	281	53.9	296	60.0
Medium smokers	310	36.2	250	39.6	200	38.4	165	33.5
Heavy smokers	101	11.8	63	10.0	40	7.7	32	6.5

## Results

Cigarette consumption, retail price and personal monthly income were used in equation (1), the OLS method, to calculate cigarette price and income elasticities. The overall cigarette price elasticity was negative, less than one, indicating that cigarette consumption or demand in Taiwan was inelastic during the study period (Table 2). Taken into consideration that cigarette price elasticity is inelastic and that reduction of the cigarette consumption is done with a strategy of raising domestic cigarette price, we speculate that cigarette prices need to be even higher, to lower consumption enough to have a clear, strong impact in improved public health outcomes. While income elasticities were not statistically different from 2000 to 2002, they did reach a statistically significant level (5%) in 2003. At that time, estimated income elasticity was positive, indicating that cigarettes were normal goods. A positive value normally means that demand for normal goods would increase as incomes rise. But the value could vary greatly among normal goods. Interestingly, in our study, we found that as incomes were rose, income elasticity became low, indicating that income increases in 2003 only had a slight effect on cigarette consumption.

**Table 2:** The estimated overall cigarette price and income elasticities of the current smokers, 2000–2003^a^

	2000		2001		2002		2003	
Price elasticity	-0.3134	(-2.894)*	-0.3684	(-3.482)*	-0.5274	(-3.143)*	-0.3090	(-1.531)
Income elasticity	0.0069	(0.708)	0.0042	(0.504)	-0.0012	(-0.125)	0.0320	(2.916)*

a. t ratios are shown in parentheses.

* p < 0.05.

After Taiwan's accession to WTO in 2002, Taiwan's first Tobacco Health and Welfare Tax added NT $5 the price of cigarettes, resulting in an increase in cigarette retail price from NT $35.2 in 2001 to NT $42.2 in 2002, about an NT $7 increase. In 2001, smokers 15 years old or older consumed an average 126.52 packs/person [14]. In 2002, the cigarette price elasticity became -0.5274, meaning that this price increase caused a reduction of cigarette consumption by 13.27 packs/person (10.5% per person) and a reduction of about 0.235 billion packs in the total consumption. Meanwhile, new cigarette consumption in 2002 was reduced to 2 billion packs for the population at and above the age of 15 in 2001. Provided there was a NT $16.8 tax on every pack of cigarettes, the tax revenue for the government would be 33.6 billion NT dollar.

However, the tax has been in force for two years, and a significant reduction in consumption has been shown to date. Cigarette taxes accounted for 40% of the retail price in 2002. Although forty percent sounds high, this proportion is still rather low when, as mentioned earlier, comparing it with the 66% or more of the retail price going for cigarettes prices in high-income countries (with the notable exception of the United States)[9]. Consequently, cigarette prices in Taiwan are well below those of many high-income countries, who have seen significant reductions in cigarette consumption. Those successes certainly impose a pressure on the government to implement another tax increase in the existing Health and Welfare Tax.

Inter-party negotiations at the Legislative Yuan resulted in changes to Tobacco and Liquor Tax Law which led to a NT $3 rise in the health tax levied on cigarettes, from NT$5 to NT$8 per pack. The cigarette industry is likely to pass the tax increase in the form of higher prices on to the consumer. A price increase of 3 NT per pack in cigarette would reflect a consumption reduction of 2.47 packs per capita, totaling 44.7 million packs, 2.2% per capita, in cigarette consumption. Cigarette consumption would be reduced to 2 billion packs (based on a population count of 2003), and the government tax revenue would be 33.46 billion, including the Health and Welfare Tax of 16 billion. We estimate that with a Health and Welfare Tax increase from 5 NT to 8 NT per pack, there would be a 6 billion NT increase in revenue from the Health and Welfare Tax.

In 2000 and 2001, the cigarette price elasticities of the current smokers were -0.3134 and -0.3684, respectively. In 2002, it had reached its maximum of -0.5274, indicating that as cigarette prices increased, so did the price elasticity. Consumers responded to the higher prices by cutting consumption. Then in 2003, after Taiwan's accession to WTO, cigarette price elasticity then lowered to -0.309, indicating that the effect of a cigarette price hike can diminish over time.

Two sets of previous data concerning the cigarette consumption, retail price and personal monthly income of the current smokers obtained during 2000–2001 and during 2002–2003, were made available to us. Using the OLS method and the rise of cigarette price in 2002 as the baseline, we computed the cigarette price and income elasticities for these two sets of data. Table 3 shows the differences in the overall cigarette price elasticity of the current smokers between before and after Taiwan's accession to WTO in 2002: before -0.4062 and after -0.3352. Our result clearly indicated that smokers were more responsive to the price after the cigarette price rose in 2002–2003. Yet, -0.4062 and -0.3352 also revealed that there was no big change in cigarette price elasticity before and after Taiwan's accession to WTO. The reason for this might be that the rise of average cigarette price was only NT $7 per pack. We speculate that a possible continuous and substantial rise in cigarette prices in the future might increase the overall price elasticity, which in turn could allow for a more effective use of the tax increases or price increases to control tobacco.

**Table 3:** The estimated cigarette price and income elasticities of different types of current smokers during 2000–2001 and during 2002–2003^a^

	2000–2001				2002–2003			
		
Characteristics	Price elasticity		Income elasticity		Price elasticity		Income elasticity	
**Overall**	-0.3352	(-4.355)*	0.0055	(0.849)	-0.4062	(-3.102)*	0.0174	(2.331)*
**Gender**								
Male	-0.3107	(-4.036)*	0.0020	(0.291)	-0.3930	(-2.935)*	0.0194	(2.450)*
Female	-0.1223	(-0.312)	-0.0206	(-0.820)	-0.1406	(-0.251)	-0.0476	(-1.903)
**Age**								
17–24	-0.4022	(-0.645)	0.0368	(1.151)	-0.1057	(-0.144)	0.0420	(1.532)
25–34	-0.0528	(-0.274)	-0.0038	(-0.227)	0.2300	(0.734)	0.0315	(1.822)
35–44	-0.1835	(-1.425)	-0.0028	(-0.206)	-0.2154	(-1.023)	-0.0060	(-0.422)
45–54	-0.1540	(-1.087)	0.0048	(0.388)	-0.4100	(-1.624)	-0.0107	(-0.724)
55-	-0.2453	(-1.073)	-0.0080	(-0.608)	-0.0475	(-0.118)	0.0008	(0.044)
**Education**								
College and above	-0.1070	(-0.514)	-0.0133	(-0.584)	-0.7007	(-2.047)*	0.0919	(4.148)*
Senior high school	-0.2772	(-2.273)*	0.0211	(1.825)	-0.5369	(-2.703)*	0.0233	(2.069)*
Junior high school	-0.5766	(-4.093)*	0.0275	(2.552)*	0.1789	(0.833)	0.0279	(2.058)*
Preliminary or lower	0.0560	(0.302)	-0.0056	(-0.440)	-0.0392	(-0.118)	-0.0191	(-1.244)
**Month income**								
No income	-0.5103	(-2.193)*			-0.8363	(-2.014)*		
<NT $20,000	-0.4570	(-2.373)*			-0.7478	(-2.316)*		
NT $ 20,000–39,999	-0.4805	(-3.953)*			-0.2861	(-1.345)		
NT $ 40,000–59,999	-0.1562	(-0.951)			-0.2624	(-1.022)		
≥ NT $ 60,000	0.2341	(1.050)			-0.1152	(-0.301)		
**Smoking degree**								
Light smokers	-0.1984	(-2.046)*	0.0087	(1.120)	-0.5320	(-3.293)*	0.0172	(1.956)
Medium smokers	-0.0228	(-0.914)	-0.0005	(-0.198)	-0.2600	(-5.068)*	-0.0046	(-1.514)
Heavy smokers	0.2573	(1.973)*	-0.0083	(-0.820)	-0.0006	(-0.005)	0.0010	(0.137)

a. t ratios are shown in parentheses.

* p < 0.05.

Cigarette price elasticity for the male smokers reached statistically significance (5%), which was higher than the price elasticity of the female smokers, indicating that men were more responsive to price elasticity than the women. The cigarette price elasticity of the male smokers in 2002–2003 was -0.393, which was higher than -0.3107 male smokers in 2000–2001. The income elasticity of male smokers in 2002–2003 was 0.0194, reaching statistical significance (5%). There was no statistical difference found in estimated cigarette price and income elasticity of smokers among the different age sub-groups, though, according to some reports in foreign countries, teenagers are more sensitive changes to cigarette prices than adults [15-17].

Education level seemed to make a difference. The price elasticity smokers with junior high school educations was -0.5766 in 2000–2001, which was higher than that of senior high school level smokers, -0.2772. In 2002-2003, the price elasticity of college level smokers was -0.7007, which was higher than that of the smokers of senior high school level, -0.5369. Therefore, between 2000 and 2001, the more educated group had a larger price elasticity, though the less educated group had the higher coefficient in 2002–2003. Our findings cover two time spans and are different from those reported by foreign researchers who have found that consumers at lower education levels respond stronger to the change of cigarette price [18]. In our study, those with no income had the greatest cigarette price elasticity, -0.5103 in 2000–2001 and -0.8363 in 2002–2003. Those with no income were more sensitive to the price rise than those with income. Light smokers had the highest cigarette price elasticity, -0.1984 in 2000–2001 and -0.532 in 2002–2003. Heavy smokers had a price elasticity of 0.2573 in 2000–2001, indicating that with the rise of cigarette price, these smokers increased their consumption of tobacco.

## Conclusions

In this study, we evaluate the effect of a new tax on the consumption of tobacco by calculating the cigarette price elasticities of various kinds of smokers and comparing those values with their reactions to an increase cigarette, and then using knowledge gained from that study, we assess the possible effect of another increase on consumption. We found that the new tax scheme implemented after Taiwan joined the WTO reduced cigarette consumption by 13.27 packs/person (10.5%). We estimated that an additional NT $3 increase in Taiwan's Health and Welfare Tax would reduce cigarette consumption here by 2.47 packs/person (2.2%).

In this study, cigarette price elasticity was less than one, meaning that in addition to reducing cigarette consumption, an additional tax would also generate additional tax revenues. Continuing price increases should reduce cigarette consumption significantly. Provided that the tax increases are proportionately larger than the resulting reduction in cigarette consumption, cigarette tax revenues will increase and can be used to reduce current National Health Insurance deficits and possibly reduce the damage and death caused by smoking related diseases.

Based on estimated cigarette price elasticities for various kinds of current smokers, we found that male smokers without income and light smokers were more sensitive to changes in cigarette prices. Teenagers (17 – 24 years old), however, were not found to be significantly influenced by the change in cigarette price, which means it will take more than just tax increases to decrease consumption among our youth. Schools will need to commit to preventive education by inculcating the students with the knowledge of tobacco hazards. Only through early preventive education starting from their childhood can we expect to see significant reduction in cigarette consumption.

Finally, the R-squared statistics of the each empirical estimation was below 0.1 for probably sake of to calculate elasticity and exclude variables like advertisement. Future research should also attempt to include these factors with cigarette demand function.

## Competing interests

The author(s) declare that they have no competing interests.

## Authors' contributions

JML performed physical measurements, collected data, and drafted the manuscript. TCH reviewed the manuscript. CYY and SHC carried out the statistical analysis and participated in data collection. All authors read and approved the final manuscript.

## Pre-publication history

The pre-publication history for this paper can be accessed here:


